# PRMT5 activates KLF5 by methylation to facilitate lung cancer

**DOI:** 10.1111/jcmm.17856

**Published:** 2023-07-17

**Authors:** Hai Zhou, Jing Chang, Jingjian Zhang, Hongzhen Zheng, Xiang Miao, Huimin Mo, Jie Sun, Qin Jia, Guangsheng Qi

**Affiliations:** ^1^ Department of Respiratory and Critical Care Medicine Shidong Hospital of Yangpu District Shanghai China; ^2^ Department of Pulmonary and Critical Care Medicine Second Affiliated Hospital of Naval Medical University Shanghai China

**Keywords:** KLF5, lung cancer, methylation, PRMT5, proliferation, stability

## Abstract

The highly expressed oncogenic factor Krüppel‐like factor 5 (KLF5) promotes various cancerous processes, such as cell growth, survival, anti‐apoptosis, migration and metastasis, particularly in lung cancer. Nevertheless, the modifications to KLF5 after translation are poorly understood. Protein arginine methyltransferase 5 (PRMT5) is considered as an oncogene known to be involved in different types of carcinomas, including lung cancer. Here, we show that the expression levels of PRMT5 and KLF5 are highly expressed lung cancer. Moreover, PRMT5 interacts with KLF5 and facilitates the dimethylation of KLF5 at Arginine 41 in a manner that depends on methyltransferase activity. Downregulation or pharmaceutical suppression of PRMT5 reduces the expression of KLF5 and its downstream targets both in vitro and in vivo. Mechanistically, the dimethylation of KLF5 by PRMT5 promotes the maintenance and proliferation of lung cancer cells at least partially by stabilising KLF5 via regulation of the Akt/GSK3β signalling axis. In summary, PRMT5 methylates KLF5 to prevent its degradation, thereby promoting the maintenance and proliferation of lung cancer cells. These results suggest that targeting PRMT5/KLF5 axis may offer a potential therapeutic strategy for lung cancer.

## 1 INTRODUCTION

Lung cancer is a major global health issue and is responsible for a significant number of cancer‐related deaths, accounting for about 18% of all cancer deaths.^1^, ^2^, ^3^ Smoking is the primary risk factor for lung cancer, but exposure to air pollution, radon gas and occupational hazards can also increase the risk. Despite advances in diagnosis and treatment, including improved imaging techniques and targeted therapies, lung cancer remains challenging to treat, particularly in advanced stages. Therefore, prevention and screening are essential for reducing the incidence and mortality of lung cancer.

Kruppel‐like factor 5, also known as KLF5, is a transcription factor that is expressed in various tissues and plays crucial roles in cell proliferation, differentiation and survival.^4^ However, abnormal expression of KLF5 has been observed in many different types of cancer, including breast,^5^ prostate,^6^ colon,^7^ ovarian cancer,^8^ liver^9^ and lung.^10^ In some cases, KLF5 overexpression has been linked to unfavourable outcomes such as increased tumour growth, poor prognosis and resistance to chemotherapy. Despite its potential as a therapeutic target, the function of KLF5 in cancer is complex and context‐dependent, especially in lung cancer. Studies have shown that KLF5 is overexpressed in lung cancer and may contribute to its development and progression.^11^ In particular, KLF5 has been shown to promote cell proliferation and invasion in lung cancer cells.^10^ It has also been associated with resistance to chemotherapy and radiation therapy in lung cancer. While KLF5 has been identified as a potential therapeutic target in lung cancer, further research is needed to fully understand its function and potential as a treatment target in this disease.

Protein arginine methyltransferase 5 (PRMT5) is a member of the Type II PRMT family that catalyses the transfer of methyl groups from S‐adenosylmethionine (SAM) to arginine residues on a substrate protein, resulting in symmetric mono‐ or di‐methylation of the substrates, which is engaged in various cellular processes, including gene expression, RNA splicing, metabolism, proliferation and DNA repair.^12^, ^13^, ^14^, ^15^ Studies have shown that overexpression of PRMT5 is associated with the development and progression of various types of cancer, including lung, breast, prostate and liver.^16^ PRMT5 promotes cancer cell growth and survival by regulating the expression of various oncogenes and tumour suppressor genes. It also plays a crucial role in forming cancer stem cells, which are responsible for tumour initiation, progression and recurrence. Exploring PRMT5 as a potential therapeutic target for cancer treatment is an active area. Several small molecule inhibitors of PRMT5 have been developed and are currently being tested in preclinical and clinical studies.^17^, ^18^ These inhibitors have shown promising results in suppressing tumour growth and inducing cancer cell death in various types of cancer. Thus, targeting PRMT5 may offer a novel and effective approach to cancer therapy.

In this study, we uncovered that PRMT5 functions as an arginine methyltransferase for KLF5 on arginine 41, leading to increased stability of the KLF5 protein in lung cancer. Furthermore, PRMT5 promotes the proliferation and growth of lung cancer cells, which can be inhibited by the specific PRMT5 inhibitors or shRNA both in vitro and in vivo. These results suggest that targeting PRMT5/KLF5 may represent a promising therapeutic strategy for lung cancer.

## 2 MATERIALS AND METHODS

### 2.1 Cell culture and chemicals

The ASTC‐a‐1 cells were obtained from the Department of Medicine, Jinan University in Guangzhou, China. All other cell lines were purchased from The Cell Bank of Type Culture Collection of the Chinese Academy of Sciences (CAS) in Shanghai. All cell lines were cultured in Dulbecco's modified Eagle's medium (DMEM, Gibco, Thermo Fisher Scientific) supplemented with 10% foetal bovine serum (FBS, Sigma cat# F2442), 50 units/mL penicillin and 50 mg/mL streptomycin. Prior to experimentation, all cell lines were tested for mycoplasma using the PlasmoTest™‐Mycoplasma Detection Kit (InvivoGen, China). The cells were incubated at 37°C with 5% CO_2_. The PRMT5‐specific inhibitor GSK3326591 (cat# SML‐1751) and cycloheximide (cat# C4859) were purchased from Sigma. Lysosome inhibitor bafilomycin A1 (BAF‐A1, cat# HY‐100558), GSK3326595 (cat#, HY‐101563) and MG‐132 (cat#, HY‐13259) were purchased from MedChemExpress (MCE).

### 2.2 Plasmids

To knock down endogenous PRMT5 and KLF5, the specific shRNAs with targeting sequences were designed: PRMT5‐shRNA1 (5′–GGATAAAGCTGTATGCTGT–3′), PRMT5‐shRNA2 (5′–GCCATCTATAAATGTCTGCTA–3′) and KLF5‐shRNA (5′–CCCTGAGTTCACCAGTATATT–3′). The scramble‐shRNA, PRMT5‐shRNAs or KLF5‐shRNA was cloned into the lentivirus vector.^19^ The human WT‐PRMT5 and a dead enzyme mutant PRMT5 cDNA were also cloned into the Flag vector (Addgene, cat# 52535) as previously described.^20^, ^21^ The human WT‐KLF5 plasmid was obtained from Addgene (cat# 40900) and cloned into the pcDNA3.1‐HA vector (Addgene, cat# 128034). Furthermore, the HA‐KLF5 point mutants were generated using the Quick‐change Lightning Site‐Directed Mutagenesis Kit (Agilent, cat# 210518), following the manufacturer's protocol.

### 2.3 Bioinformatics analysis

RNA‐sequencing expression (level 3) profiles and corresponding clinical information for PRMT5 and KLF5 were downloaded from the TCGA dataset (https://www.cancer.gov/ccg/research/genome‐sequencing/tcga). R software GSVA package was used to analyse, choosing parameter as method = 'ssgsea'. The correlation between genes and pathway scores was analysed by Spearman correlation. All the analysis methods and R packages were implemented by R version 4.0.3. *p*‐value <0.05 was considered statistically significant.

### 2.4 PRMT5 and KLF5 stable depletion cell lines

To establish stable knockdown of PRMT5 and KFL5 cell lines, 293T cells were co‐transfected with lentiviral plasmids containing either scramble shRNA, PRMT5‐specific shRNAs or KLF5‐shRNA, along with helper plasmids MD2G and PAX2, using Lipofectamine™ 2000 transfection reagent as per the manufacturer's instructions. After 24 h, the media was replaced with fresh media, and the harvested media was filtered and titred after 48 h. Next, cells were infected with equal amounts of lentivirus containing scramble‐shRNA, PRMT5‐shRNAs or KLF5‐shRNA. Following 24 h of infection, cells were selected with puromycin (Sigma, cat# p9620) for 48 h to generate stable knockdown cells.

### 2.5 Gene expression

To determine gene expression levels, quantitative real‐time PCR (qRT‐PCR) was performed. Total RNA was extracted from cells using TRIzol reagent (Invitrogen, cat# 15596‐018) following the manufacturer's protocol. The concentration of the extracted RNA was measured using NanoDrop 2000 spectrophotometers, and 1 μg of total RNA was used for reverse transcription using the C1000 Touch PCR Thermal Cycler (Bio‐Red). The expression of target genes was quantified using qRT‐PCR with the ABI7500 PCR machine (Applied Biosystems™) and SYBR green fluorescent dye (Bio‐Rad, cat# 1725272). The following primers were used in this study: human PRMT5 forward: 5′–CCTGTGGAGGTGAACACAGT–3′ and revise: 5′–AGAGGATGGGAAACCATGAG–3′; human KLF5, forward: 5′–ACACCAGACCGCAGCTCCA–3′ and revise: 5′–TCCATTGCTGCTGTCTGATTTGTAG−3′; human Slug, forward: 5′–CGAACTGGACACACATACAGTG–3′ and revise: 5′–CTGAGGATCTCTGGTTGTGGT–3′; human FGF‐BP1, forward: 5′–TGTTCAGAGGCTGTTTCCTG–3′ and revise: 5′–TTCAGCAGAAAGTTCGTTGC–3′; human GAPDH, forward: 5′–TGTGGGCATCAATGGATTTGG–3′ and revise: 5′–ACACCATGTATTCCGGGTCAAT–3′. GAPGH served as an internal control. The relative mRNA expression level was calculated by the method of ΔΔ‐Ct.

### 2.6 Cell viability assay

To conduct the cell proliferation assay, we seeded the A549 and ASTC‐a‐1 cells in 96‐well plates and cultured them with either a vehicle or increasing concentrations of GSK591. After incubation, we added 20 μL of CellTiter 96 AQueous One Solution (Promega Corporation, Madison, WI. cat# G3582) to each well and incubated the plates for 2 h. We then used an Infinite 200 plate reader (TECAN, Mönnedorf, Switzerland) to measure the absorbance at OD490. To determine the background values, we included wells that contained only the culture medium.

### 2.7 Immunofluorescence

A549 cells were seeded into 6‐well plates and allowed to adhere for 24 h before treatments. The cells were fixed for 10 min at room temperature and then permeabilised with ice‐cold methanol at −20°C for 15 min. After permeabilisation, the cells were washed twice with PBS and blocked for 60 min at room temperature in a blocking buffer (5% normal goat serum). The primary antibodies, including KLF5 (Cell Signalling Technology, cat# 40674) and β‐actin (Santa Cruz Biotechnology, cat# sc‐47778) were diluted 1:100 in blocking buffer and incubated with the cells overnight at 4°C. After incubation with the primary antibodies, the cells were washed three times with PBS and then incubated with Alexa Fluor 594‐conjugated goat anti‐mouse secondary antibody (Thermo Fisher, cat# A‐11005) and Alexa Fluor 488‐conjugated goat anti‐rabbit secondary antibody (Thermo Fisher, cat# A‐11034). Before imaging, the nuclei were stained with DAPI (Sigma, cat# D9542) for 30 min. The images were acquired using a confocal microscopy system (LSM700, Zeiss) and analysed.

### 2.8 Western blotting

Western blot analysis was conducted using the previously described method.^22^ Briefly, the cells were lysed using the RIPA buffer (Cell Signalling Technology, cat# 9806). The lysates were centrifuged at maximum speed for 10 min at 4°C, and protein concentration was determined using the Bradford method. The proteins were then separated using sodium dodecyl sulphate/polyacrylamide gel electrophoresis (SDS/PAGE) and transferred onto PVDF membranes (Bio‐Rad, cat# 1620177). After washing the membranes three times with TBST, the membranes were blocked with 5% non‐fat milk for 1 h at room temperature. The membranes were then incubated overnight at 4°C with the specified primary antibodies: PRMT5 (Santa Cruz Biotechnology, cat# sc‐376937), KLF5 (Cell Signaling Technology, cat# 40674), phospho‐Ser473‐Akt (Cell Signaling Technology, cat# 4060), total Akt (Cell Signaling Technology, cat# 4691), Symmetric Di‐Methyl Arginine Motif [sdme‐RG] MultiMab™ (Cell Signaling Technology, cat# 13222), Slug (Cell Signaling Technology, cat# 9585), GSK3β (Cell Signaling Technology, cat# 12456), p‐Ser9‐GSK3β (Cell Signaling Technology, cat# 5558), Cyclin D1 (Cell Signaling Technology, cat# 55506), Flag tag (Sigma, cat# F7425) and β‐actin (Santa Cruz Biotechnology, cat# sc‐47778). After primary antibody incubation, the membranes were washed three times with TBST and incubated with appropriate secondary antibodies conjugated to HRP, goat anti‐rabbit (Santa Cruz Biotechnology, cat# sc‐2004) or goat anti‐mouse (Santa Cruz Biotechnology, cat# sc‐2005), at room temperature for 2 h. The proteins were visualized using SuperSignal West Pico Chemiluminescent Substrate western blotting reagents (Thermo Fisher Scientific, cat# 34580).

### 2.9 Co‐immunoprecipitation

To perform the co‐immunoprecipitation experiment, we seeded A549 and ASTC‐a‐1 cells in 15 cm culture dishes and lysed the cells using lysis buffer (20 mM Tris pH 7.4, 150 mM NaCl, 2 mM EDTA, 2 mM EGTA, 1 mM sodium orthovanadate, 50 mM sodium fluoride, 1% Triton X‐100, 0.1% SDS and 100 mM phenylmethylsulfonyl fluoride). Afterward, we centrifuged the lysates at maximum speed for 10 min at 4°C, and the cleared cell extracts were pre‐cleared with protein A and protein G beads (Santa Cruz Biotechnology, cat# sc‐2001 and cat# sc‐2002) at 4°C for 1 h. We then added 5°μg of primary antibodies, including PRMT5 (Santa Cruz Biotechnology, cat# sc‐376937), KLF5 (Cell Signaling Technology, cat# 51586), SYM10 (Millipore& Sigma, cat# 07‐412), HA (BioLegend, cat# 901502) or isotype IgG to the cleared cell extracts and incubated them overnight at 4°C. Next, we incubated the cell extracts with protein A or protein G beads at 4°C for 3 h, followed by three washes with wash buffer (100 mM NaCl, 50 mM Tris pH 7.5, 0.1% NP‐40, 3% glycerol and 100 mM phenylmethylsulfonyl fluoride). Finally, we eluted the beads‐bound proteins by adding 1× Laemmli sample buffer and heating at 95°C for 10 min. We analysed the protein–protein interactions using western blotting.

### 2.10 Xenograft mouse model and in vivo tumourigenesis analysis

The animal study was approved by the Ethical Committee of Shanghai Shidong Hospital. For in vivo tumourigenesis study, we used nude male mice that were 8 weeks old. A total of 1 × 10^6^ A549 cells were suspended in Matrigel and PBS (a total volume of 0.2 mL) with a 1:3 ratio and then injected into the mice. The tumour size was monitored every 2 days. After 1 week, mice with tumours of similar size were randomly divided into two groups: a vehicle control group and a GSK595 treatment group (100 mg/kg). The animals were treated daily for 2 weeks with either vehicle or GSK595 (100 mg/kg). Alternatively, the same number of PRMT5 depletion A549 cells or control cells at the density of 1 × 10^6^ were suspended in Matrigel and PBS (a total volume of 0.2 mL) with a 1:3 ratio and then injected into the mice. The tumour size was monitored every 2 days. Finally, the mice were sacrificed on Day 25, and the tumours were harvested for further analysis.

### 2.11 Statistical analysis

The experiments were performed in triplicate under the same conditions, and the results were presented as means ± standard error (SE). Statistical analysis was conducted using one‐way anova (GraphPad Software, Inc., La Jolla, CA) or unpaired *t*‐test (Microsoft Excel), as specified in the figure legend. Statistical significance was defined as *p* < 0.05.

## 3 RESULTS

### 3.1 KLF5 and PRMT5 are highly expressed in lung cancer cells

**FIGURE 1:**
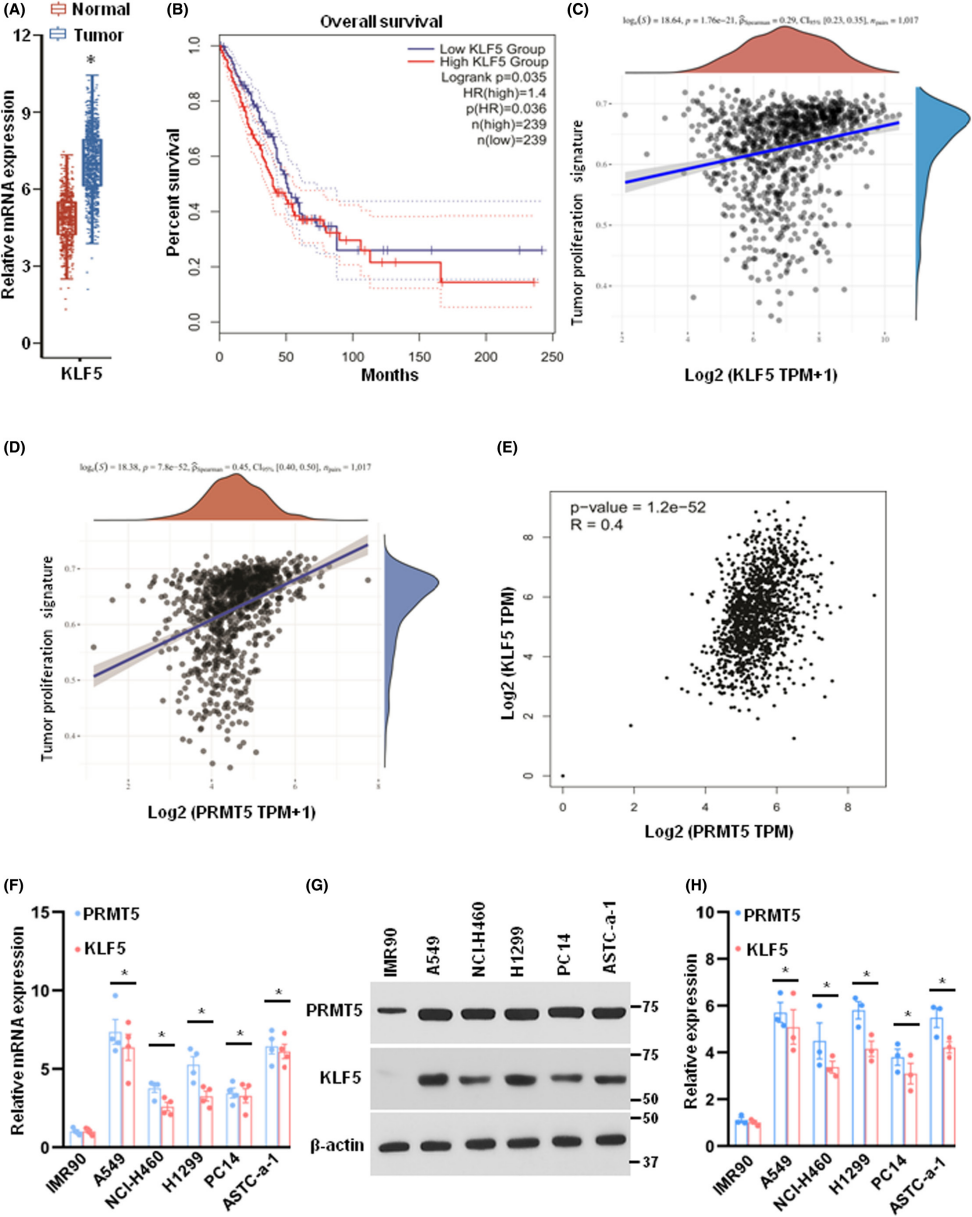
PRMT5/KLF5 is overexpressed in lung cancer cells. (A) The relative expression of KLF5 in lung cancer tissues (*n* = 1017) and normal tissues (*n* = 627). *p < 0.05 versus normal. (B) KLF5 expression level was negatively correlated with the patient's overall survival with TCGA database analysis. *n* = 239 for KLF5‐low group and KLF5‐high group. *p* values were determined by log‐rank test and *p* = 0.035. (C and D) The correlations between individual gene and pathway score was analysed with Spearman method. The abscissa represents the distribution of the gene expression, and the ordinate represents the distribution of the pathway score. The density curve on the right represents the trend in distribution of pathway immune score; the upper density curve represents the trend in distribution of the gene expression. The value on the top represents the correlation *p* value, correlation coefficient and correlation calculation method. (E) The correlations between PRMT5 and KLF5 were analysed with Spearman method. (F) The mRNA expression of PRMT5 and KLF5 were measured by qRT‐PCR in different lung cancer cell lines. *p < 0.05 versus IMR90 cells. G and H. The protein expression of PRMT5 and KLF5 were detected by western blotting in different lung cancer cell lines. *p < 0.05 versus IMR90 cells.

To explore the expression and potential function of KLF5 and PRMT5 in lung cancer, we first evaluated the mRNA expression of KLF5 in lung cancer tissues and matched normal tissues. As shown in Figure 1A, KLF5 mRNA expression was significantly higher in lung cancer tissues compared to the matched normal tissues. This abnormal expression level was closely related to the survival rate. As shown in Figure 1B, the patients with high expression levels of KLF5 had a low survival rate compared with those with low expression levels of KLF5. Previous studies have shown that PRMT5 was overexpressed in lung cancer and played a key role in governing the EMT and metastasis.^23^ We then assessed the tumour proliferation signature in lung cancer and found that both KLF5 and PRMT5 were positively correlated with tumour proliferation signature (Figure 1C,D), suggesting a potential relationship between these two genes. Further gene correlation analysis revealed a positive correlation between PRMT5 and KLF5 expression (Figure 1E). To confirm our findings, we assessed the mRNA and protein expression levels of PRMT5 and KLF5 in lung cancer cell lines and normal human foetal lung fibroblast cells (IMR90). Consistent with our previous results, we observed significantly higher expression of both PRMT5 and KLF5 in lung cancer cell lines compared to IMR90 (Figure 1F–H). These findings suggest that PRMT5 and KLF5 may cooperate to regulate lung cancer development and growth.

### 3.2 PRMT5 methylates KLF5 through direct interaction in lung cancer cells

**FIGURE 2:**
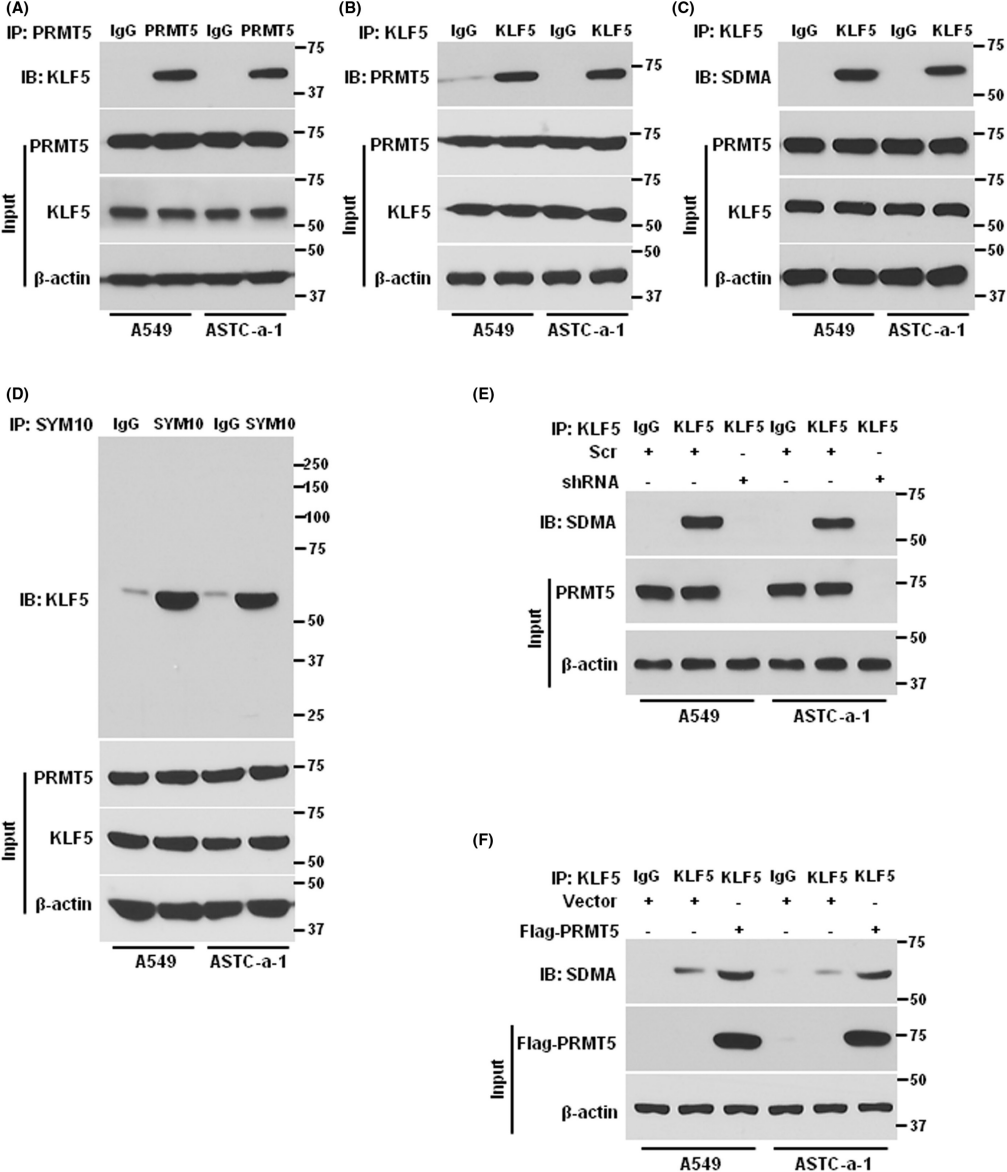
PRMT5 directly methylates KLF5. (A) The interaction between PRMT5 and KLF5 was captured through co‐immunoprecipitation (Co‐IP) technique. Specifically, the lysate was immunoprecipitated using an anti‐PRMT5 antibody, and then immunoblotted with an anti‐KLF5 antibody in both A549 and ASTC‐a‐1 cells. (B) The Co‐IP was performed using an anti‐KLF5, and then immunoblotted with an anti‐PRMT5 antibody in both A549 and ASTC‐a‐1 cells. (C) The endogenous KLF5 methylation was detected by Co‐IP. The lysate was immunoprecipitated using an anti‐KLF5 antibody, and then immunoblotted with an anti‐SDMA antibody in both A549 and ASTC‐a‐1 cells. (D) The Co‐IP was performed using an anti‐SYM10 antibody, and then immunoblotted with an anti‐KLF5 antibody in both A549 and ASTC‐a‐1 cells. (E) A549 and ASTC‐a‐1 cells were infected with lentivirus containing scramble control or PRMT5‐shRNA1. The lysate was immunoprecipitated using an anti‐KLF5 antibody, and then immunoblotted with an anti‐SDMA antibody. (F) The cell in (E) were transfected with vector or Flag‐PRMT5. The lysate was immunoprecipitated using an anti‐KLF5 antibody, and then immunoblotted with an anti‐SDMA antibody.

To discover whether PRMT5 interacts with KLF5 in human lung cancer cells, we used the immunoprecipitation (IP) method to detect the endogenous interaction between PRMT5 and KLF5. As shown in Figure 2A, PRMT5 protein directly interacted with KLF5 via immunoprecipitation with PRMT5 antibody in both A549 and ASTC‐a‐1 cells. Further, KLF5 protein directly interacted with PRMT5 via immunoprecipitation with KLF5 antibody (Figure 2B). These results strongly imply that PRMT5 may methylate KLF5. We subsequently tested whether PRMT5 may directly methylate KLF5. To this end, we pulled down KLF5 protein with the specific KLF5 antibody and the methylation was detected by SDMA antibody. As shown in Figure 2C, as expected, KLF5 was methylated in both A549 and ASTC‐a‐1 cells, which was further validated via IP using symmetric di‐methylated arginine (SYM10) antibody (Figure 2D). To further confirm our hypothesis, we generated the PRMT5 stable depletion cells and then measured the methylation of KLF5. As shown in Figure 2E, KLF5 was methylated in scramble control cells, whereas the methylation was almost disappeared in PRMT5 depletion cells. We next reintroduced the PRMT5 in PRMT5 depletion cells and the KLF5 methylation was evaluated in both A549 and ASTC‐a‐1 cells. As shown in Figure 2F, the methylation of KLF5 was very weak in PRMT5 depletion cells, while the methylation was strongly enhanced when PRMT5 was overexpressed. Altogether, our findings indicate that PRMT5 directly interacts with and methylates KLF5 in human lung cancer cells.

### 3.3 PRMT5 methylates KLF5 at Arginine 41 and regulates its stability

**FIGURE 3:**
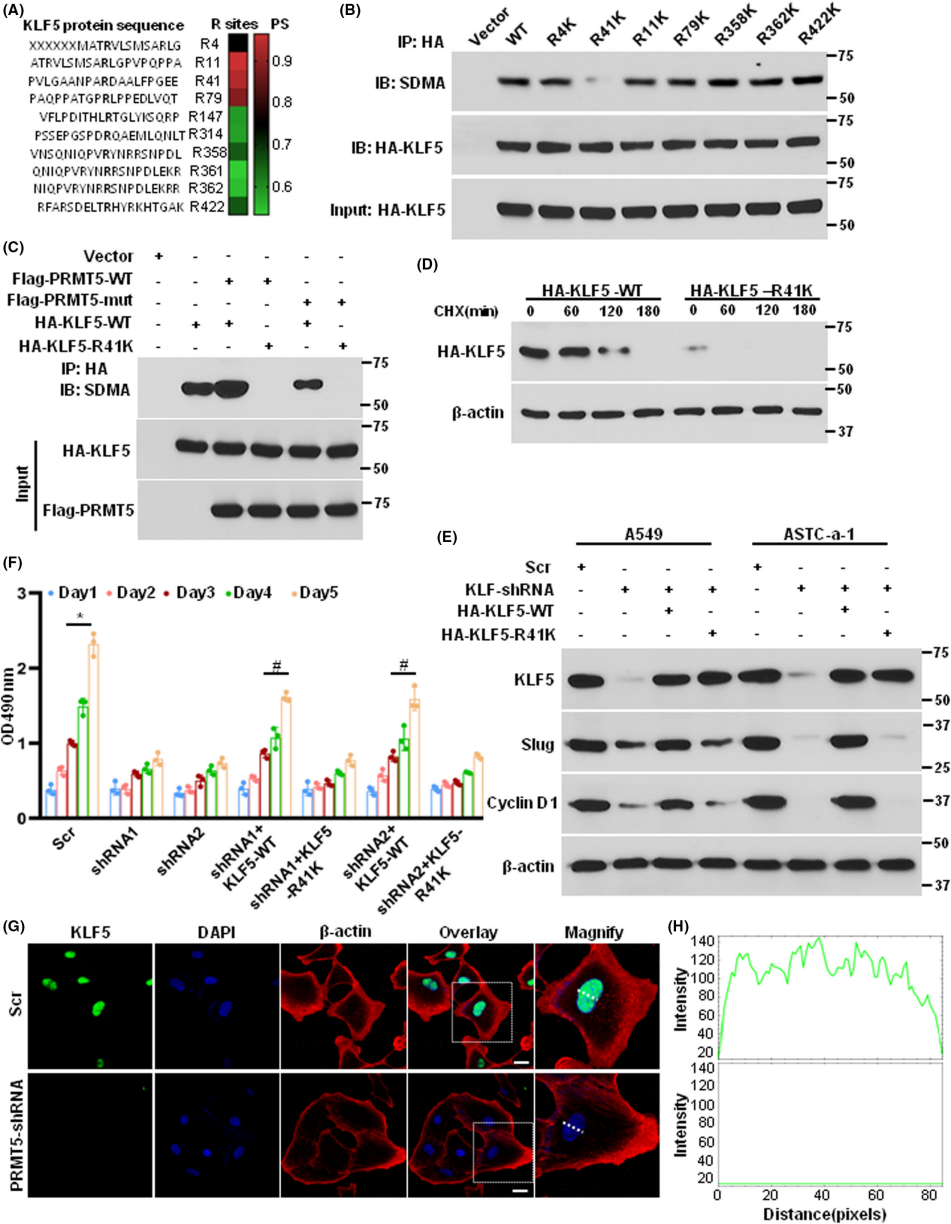
PRMT5 methylates KLF5‐R41 and controls its stability. (A) Prediction of the potential arginine sites in KLF5 for the methylation by PRMT5. PS means prediction score. Ten potential arginine sites in KLF5 were shown. (B) A549 cells were transfected with indicated HA‐tagged KLF5 and the proteins were pull‐down with HA antibody. The methylation was evaluated by western blotting using SDMA antibody. (C) A549 cells were transfected with indicated plasmids and the proteins were pull‐down with HA antibody. The methylation was evaluated by western blotting using SDMA antibody. (D) A549 cells were transfected with HA‐tagged KLF5‐WT or HA‐tagged KLF5‐R41K and then were treated with CHX at the indicated time points. The KLF5 stability was detected by western blotting. (E) A549 and ASTC‐a‐1 cells were infected with scramble (scr) shRNA or KLF5‐shRNA and then the KLF5 depletion cells were transfected with HA‐tagged KLF5‐WT or HA‐tagged KLF5‐R41K. The KLF5 and its downstream targets were detected by western blotting. (F) A549 cells were infected with scramble (scr) shRNA or PRMT5‐shRNAs and then the PRMT5 depletion cells were transfected with HA‐tagged KLF5‐WT or HA‐tagged KLF5‐R41K. Cell proliferation was measured at the indicated time points. *n* = 3, *p < 0.05 versus Scr and ^#^ *p* < 0.05 versus shRNA1 and shRNA2. (G) A549 cells were infected with scramble (scr) shRNA or PRMT5‐shRNA1 and the KLF5 expression was measured by immunofluorescence. Bar = 50 μm. *n* = 3. H. The expression of KLF5 in nucleus (the intensity of green signal) was quantified.

To identify the methylation sites induced by PRMT5 on KLF5, we utilized protein methylation tools^24^ to predict potential methylation sites on the KLF5 protein sequence. As shown in Figure 3A, 10 potential methylation sites were identified. To confirm which site was methylated by PRMT5, we generated HA‐tagged mutants by replacing arginine with lysine and detected the methylation. As shown in Figure 3B, only the KLF5‐R41K mutation significantly reduced methylation, while other mutations had no effect when equal amounts of KLF5 were pulled down. We co‐expressed PRMT5, KLF5 and the related mutants and found that overexpression of PRMT5‐WT significantly enhanced KLF5 methylation but not the enzyme activity‐deficient mutant (Figure 3C). Moreover, both PRMT5‐WT and the enzyme activity‐deficient mutant had no effect on the methylation of the KLF5‐R41K mutation, indicating that PRMT5 mainly methylates KLF5 at arginine 41 in lung cancer cells. As Fbw7 mediates KLF5 degradation via the proteasome, we explored the stability of KLF5 and the R41K mutant in lung cancer cells. As shown in Figure 3D, the KLF5 protein stability was significantly higher than that of the R41K mutant when treated with CHX at different time points. We next investigated whether the KLF5‐R41K mutation affects the expression of downstream targets of KLF5 and lung cancer cell proliferation. We generated KLF5 depletion cells and reintroduced KLF5‐WT and R41K into these cells. Figure 3E showed that the reintroduction of KLF5‐WT rescued the expression of downstream targets of KLF5, such as slug and cyclin D1, but not the R41K mutant. Moreover, the reintroduction of KLF5‐WT partially rescued cell proliferation in PRMT5 depletion cells but not the R41K mutant (Figure 3F). Finally, we used PRMT5 depletion cells to detect KLF5 expression and found that KLF5 was mainly located and expressed in the nucleus in control cells, whereas KLF5 expression was almost absent in PRMT5 depletion cells (Figure 3G,H), indicating that PRMT5 may regulate KLF5 protein stability. Collectively, our findings suggest that PRMT5 methylates KLF5 at arginine 41 to stabilize KLF5 and promote lung cancer cell growth.

### 3.4 PRMT5 governs KLF5 stability via Akt/GSK3 signalling cascades

**FIGURE 4:**
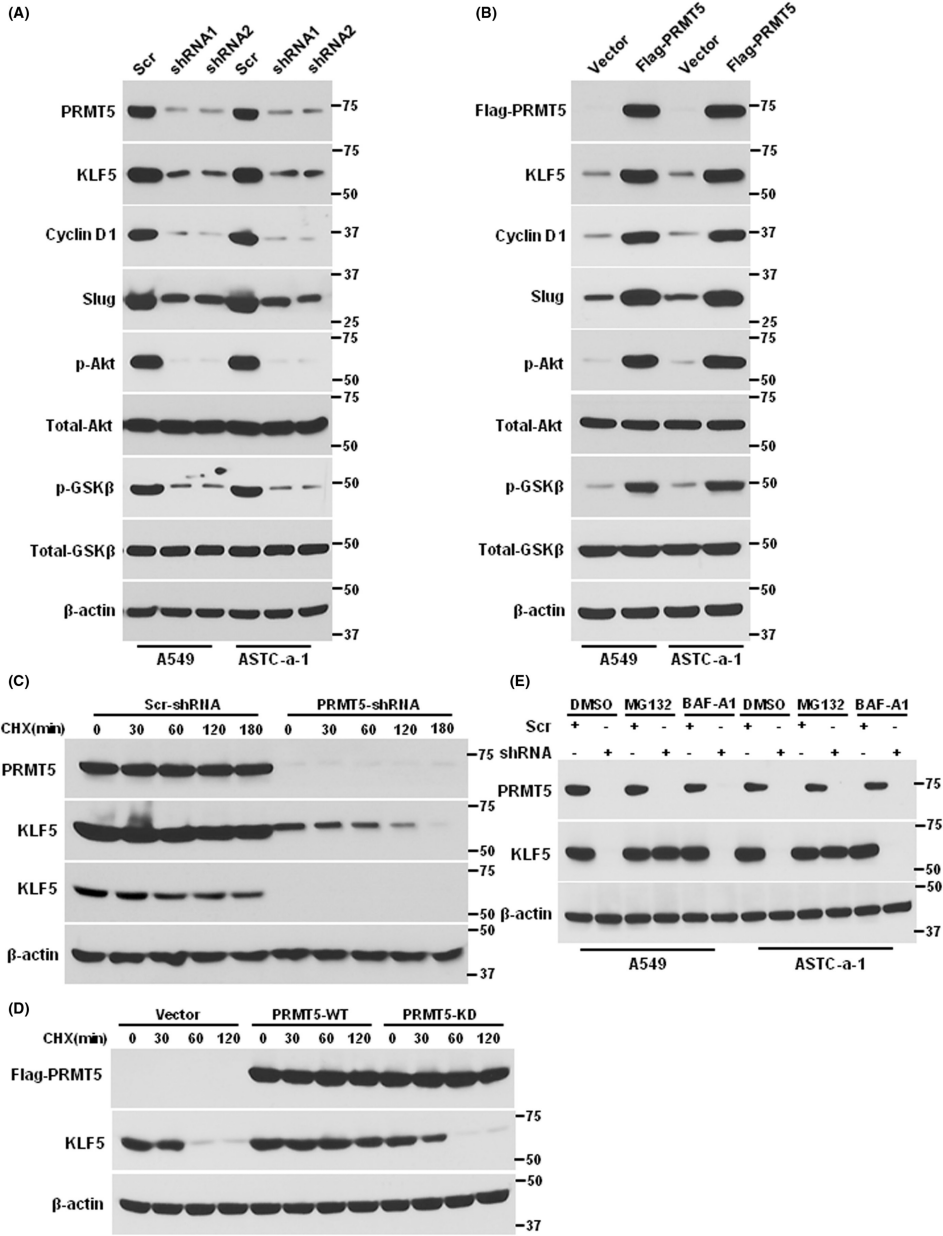
RMT5 stabilizes KLF5 via Akt/GSK3β signalling axis. (A) A549 and ASTC‐a‐1 cells were infected with lentivirus containing scramble control or PRMT5‐shRNAs and the indicated proteins were detected by western blotting. (B) The cells in (A) were transfected with vector or Flag‐PRMT5 and the indicated proteins were detected by western blotting. (C) A549 cells were infected with lentivirus containing scramble control or PRMT5‐shRNA1 following treatment with cycloheximide (CHX, 50 μg/mL) for 0, 30, 60, 120 and 180 min. The PRMT5 and KLF5 protein expression levels were detected by western blotting. The longer exposure and shorter exposure were shown. (D) A549 cells were transfected with Vector, Flag‐PRMT5 or Flag‐PRMT5‐KD for 48 h and then treated with CHX for the 0, 30, 60 and 120 min. The Flag‐PRMT5 and KLF5 protein expression levels were detected by western blotting. (E) A549 and ASTC‐a‐1 cells were infected with lentivirus containing scramble control or PRMT5‐shRNA1 following treatment with proteasome inhibitor MG132 (10 μM) or lysosome inhibitor BAF‐A1 (100 nM) for 6 h. The PRMT5 and KLF5 protein expression levels were detected by western blotting.

As PRMT5 directly interacted with and methylated KLF5, we asked whether and how PRMT5 regulated the stability of KLF5, which is entirely unknown. To achieve this, we examined the expression levels of KLF5 and its downstream targets in cells with depleted PRMT5 using western blotting. Our results indicated that PRMT5 downregulation significantly reduced the expression levels of KLF5, cyclin D1 and slug (Figure 4A), suggesting that PRMT5 plays a role in regulating KLF5 stability. Previous research has shown that GSK3β phosphorylates KLF5 and promotes its degradation. Recent studies reported that PRMT5 activates Akt by methylation, which inhibits GSK3β activity. We observed a distinct decrease in the phosphorylation of Akt and GSK3β in PRMT5‐depleted cells, indicating that PRMT5 regulates KLF5 stability via the Akt/GSK3 signalling pathway. We confirmed our hypothesis by overexpressing PRMT5‐WT in PRMT5 depletion cells, which increased the stability of KLF5 and its downstream targets, as well as the phosphorylation of Akt and GSK3β (Figure 4B). To further investigate the mechanism, we assessed the stability of KLF5 in PRMT5‐depleted cells treated with the protein synthesis inhibitor CHX. Our results showed that KLF5 stability was significantly higher in control cells than in PRMT5‐depleted cells (Figure 4C). We also overexpressed PRMT5‐WT and an enzyme activity‐deficient mutant in PRMT5 depletion cells and assessed KLF5 stability using western blotting. Our findings showed that PRMT5‐WT increased KLF5 stability, but the enzyme activity‐deficient mutant did not (Figure 4D). Finally, we investigated the mechanism by which PRMT5 mediates KLF5 stability. We found that PRMT5 downregulation reduced KLF5 expression, which was blocked by the proteasome inhibitor MG132 but not the lysosome inhibitor BAF‐A1 (Figure 4E). These results suggest that PRMT5 regulates KLF5 stability through the proteasomal degradation pathway. Overall, our findings indicate that PRMT5 plays a crucial role in regulating KLF5 stability via the Akt/GSK3β signalling pathway and proteasomal degradation pathway.

### 3.5 Pharmacological inhibition of PRMT5 represses lung cancer cell growth

**FIGURE 5:**
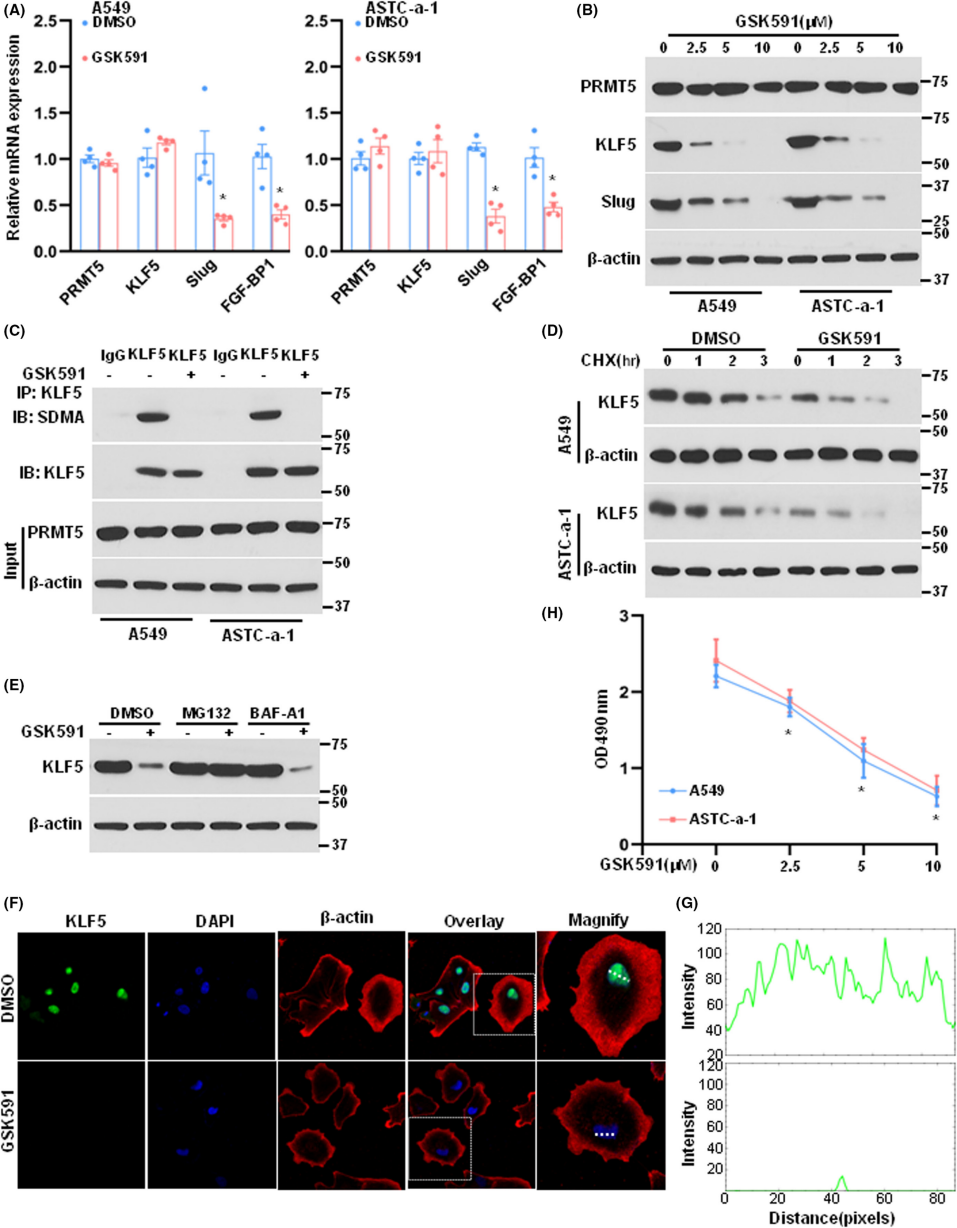
Pharmacological blocking PRMT5 represses lung cancer cell growth. (A) A549 and ASTC‐a‐1 cells were treated with vehicle or GSK591 and the gene expression was analysed with qRT‐PCR. *n* = 4. *p < 0.05 versus DMSO. (B) A549 and ASTC‐a‐1 cells were treated with vehicle or GSK591 for indicated concentrations and the protein expression levels of PRMT5, KLF5 and slug were detected by western blotting. (C) A549 and ASTC‐a‐1 cells were treated with vehicle or GSK591 and then the lysate was immunoprecipitated using an anti‐KLF5 antibody. The methylation was evaluated by western blotting using SDMA antibody. (D) A549 and ASTC‐a‐1 cells were treated with vehicle or GSK591 and then treated with CHX for indicated time points. The KLF5 protein expression was evaluated by western blotting. (E) A549 cells were treated with vehicle or GSK591 and then treated with proteasome inhibitor MG132 (10 μM) or lysosome inhibitor BAF‐A1 (100 nM) for 6 h. The KLF5 protein expression levels were detected by western blotting. F. A549 cells were treated with vehicle or GSK591 and the KLF5 expression was measured by immunofluorescence. Bar = 50 μm. *n* = 3. (G) The expression of KLF5 in nucleus (the intensity of green signal) was quantified. (H) A549 and ASTC‐a‐1 cells were treated with vehicle or GSK591 at the different concentrations and cell proliferation was measured. *n* = 6. *p < 0.05 versus control.

We investigated whether GSK591, a specific inhibitor of PRMT5, could control KLF5 expression and proliferation in lung cancer cells, as PRMT5 has emerged as a promising therapeutic target for cancer treatment, and several specific small‐molecule inhibitors of PRMT5 have been developed. First, we evaluated the mRNA expression levels of PRMT5, KLF5 and its targets. Our findings showed that PRMT5 and KLF5 mRNA expression levels were unchanged when treated with GSK591, whereas cyclin D1 and slug mRNA expression levels were decreased, indicating that PRMT5 regulates KLF5 expression at the post‐translational level rather than the transcriptional level (Figure 5A). Furthermore, KLF5 and slug protein expression levels were reduced in a dose‐dependent manner upon GSK591 treatment (Figure 5B). Treatment with GSK591 also blocked the methylation of KLF5 by PRMT5 in lung cancer cells (Figure 5C). To further investigate the effects of GSK591, we assessed the stability of KLF5 upon treatment of GSK591 in the presence of the protein synthesis inhibitor CHX. Our results showed that KLF5 stability was significantly higher in control cells than in GSK591‐treated cells (Figure 5D). We also confirmed that inhibition of PRMT5 reduced KLF5 expression, which was blocked by the proteasome inhibitor MG132 but not the lysosome inhibitor BAF‐A1 (Figure 5E). Additionally, KLF5 was predominantly located and expressed in the nucleus in control cells, whereas KLF5 expression was almost absent in GSK591‐treated cells (Figure 5F,G). Finally, inhibition of PRMT5 by GSK591 suppressed lung cancer cell proliferation in a dose‐dependent manner (Figure 5H).

### 3.6 Inhibition or downregulation of PRMT5 impairs tumour growth in nude mice

**FIGURE 6:**
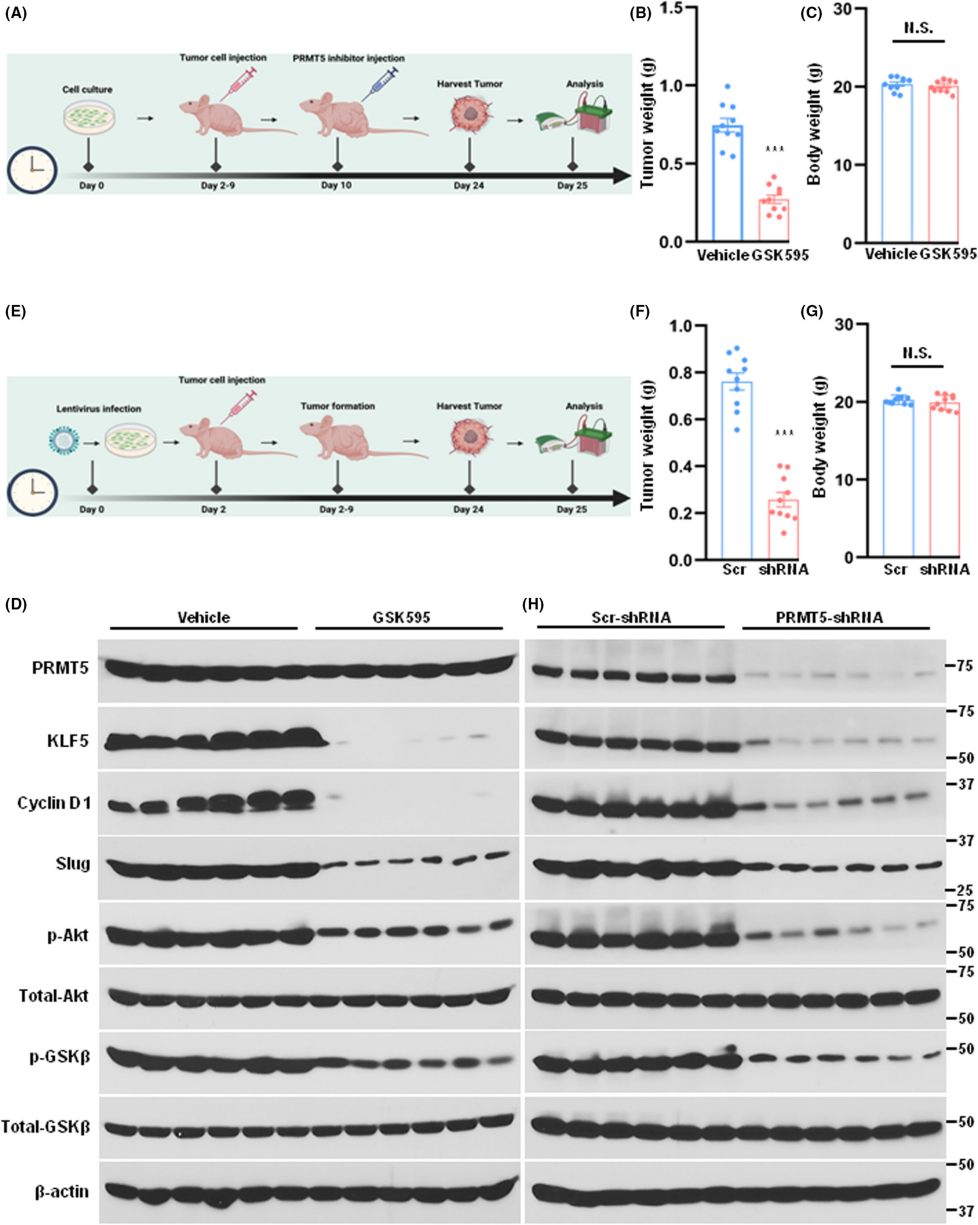
Inhibition of PRMT5 suppresses tumour growth in nude mice. (A) Schematic diagram of in vivo study using the PRMT5 inhibitor GSK3326595 (GSK595) in a xenograft model. (B) Tumour weights were measured in both the vehicle control group and the GSK595 treatment group (*n* = 10, per group). The results showed a statistically significant difference in tumour weight between the two groups (***p < 0.001 vs. vehicle control group). (C) Body weights were measured in both the vehicle control group and the GSK595 treatment group (*n* = 10, per group). The results showed no difference between the two groups (N.S. means not significant). (D) The indicated proteins were detected by western blotting in primary tumour samples (*n* = 6, per group). (E) Schematic diagram of in vivo study using the PRMT5 depletion cells in a xenograft model. (F) Tumour weights were measured in both the scramble control group and the PRMT5‐shRNA group (*n* = 10, per group). The results showed a statistically significant difference in tumour weight between the two groups (***p < 0.001 vs. scramble control group). (G) Body weights were measured in both the scramble control group and the PRMT5‐shRNA group (*n* = 10, per group). The results showed no difference between the two groups (N.S. means not significant). (H) The indicated proteins were detected by western blotting in primary tumour samples (*n* = 6, per group).

We next conducted an investigation to determine whether the administration of GSK595 or silencing PRMT5 could inhibit tumour growth in vivo. For this purpose, a xenograft model was applied, and the procedures were shown in Figure 6A,E. As expected, tumour growth was remarkably repressed upon treatment of GSK595 or silencing PRMT5 (Figure 6B,F), while no statistically significant difference in body weight was observed among the various groups of those node mice (Figure 6C,G). Finally, we examined the levels of PRMT5, KLF5, cyclin D1, slug, p‐Akt and p‐GSK3β expression in the primary tumour samples and observed that inhibition or downregulation of PRMT5 led to a reduction in the protein expression of these markers that were closely related to the epithelial–mesenchymal transition (EMT) and metastasis (Figure 6D,H). Taken together, our molecular analysis revealed a novel mechanism underlying the regulation of KLF5 protein expression and function in lung cancer, which involves arginine methylation by PRMT5 and proteasome‐mediated degradation.

## 4 DISCUSSION

**FIGURE 7:**
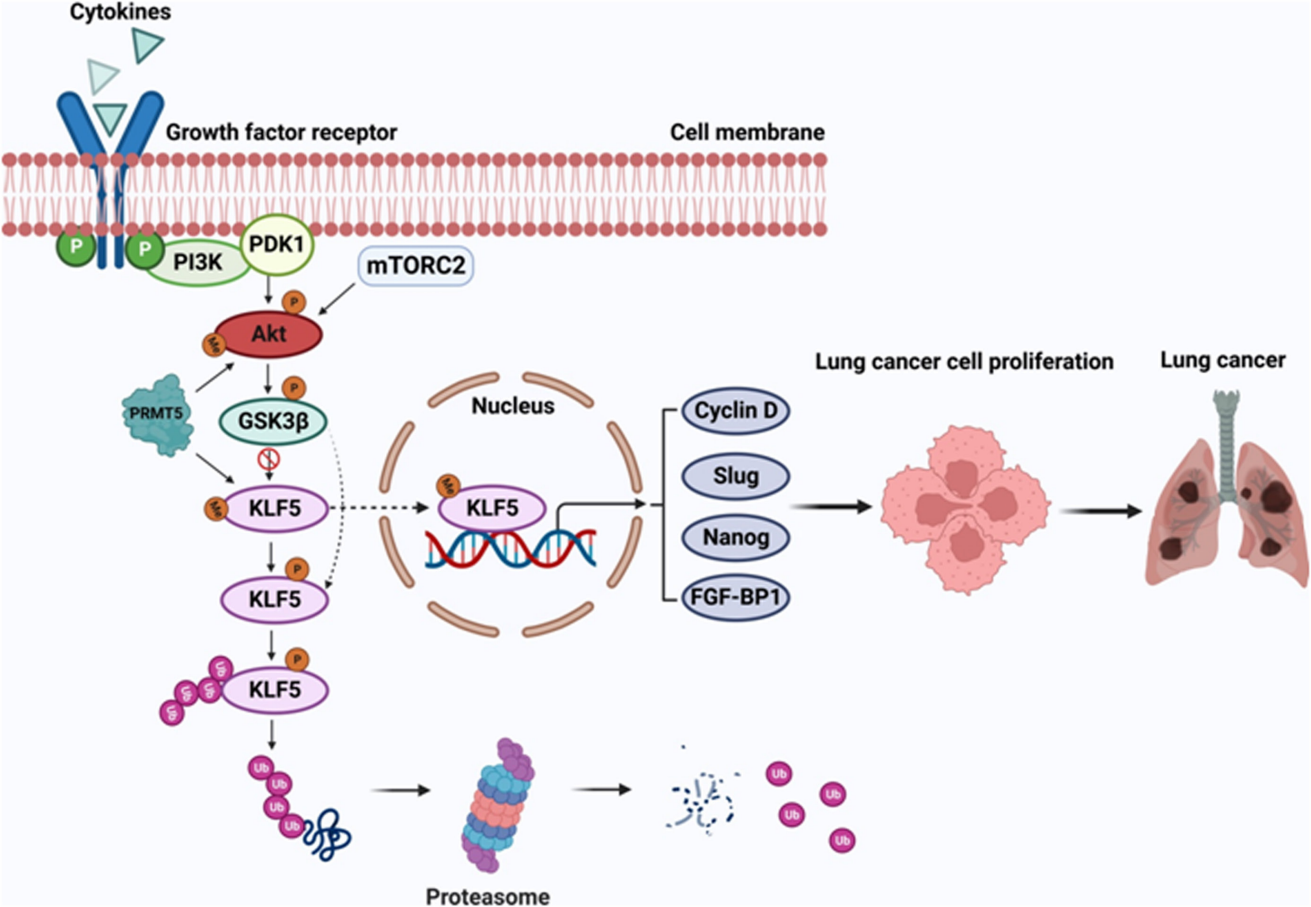
Graphic depicts the proposed mechanism by which PRMT5 regulates KLF5 to promote lung cancer cell proliferation and tumourigenesis. In this model, PRMT5 methylates KLF5 at the residue of arginine 41, leading to its stabilisation and activation. This, in turn, enhances the transcription of downstream targets involved in cell proliferation, migration and invasion, ultimately promoting tumour growth and metastasis. The stabilisation of KLF5 and upregulation of KLF5 downstream targets, such as CyclinD1, slug, Nanog and FGF‐BP1 may also contribute to the observed resistance to chemotherapy and radiation therapy in lung cancer cells. Therapeutic targeting of the PRMT5/KLF5 axis may represent a promising strategy for the treatment of lung cancer.

More and more evidence indicates that PRMT5 is a carcinogenic protein that plays a pivotal role in various human cancers,^25^ especially in lung cancer.^26^ Nevertheless, how PRMT5 regulates lung cancer development, progression and downstream targets are largely unknown. In the present study, we found that both PRMT5 and KLF5 were highly expressed and closely correlated in lung cancer cells (Figure 1). Further investigation showed that PRMT5 directly interacted with and methylated KLF5 in human lung cancer cells (Figure 2). Moreover, PRMT5 methylated KLF5 at Arginine 41 to regulate its stability through Akt/GSK3β signalling axis (Figures 3 and 4). Finally, we found that inhibition or downregulation of PRMT5 resulted in repressing lung cancer cell growth both in vitro and in vivo (Figures 5 and 6). These findings indicate that targeting PRMT5/KLF5 axis (Figure 7) may offer a potential therapeutic strategy for lung cancer.

Non‐small‐cell lung cancer (NSCLC) is the most aggressive subtype of lung cancer and has the poorest prognosis.^27^ Previous studies have demonstrated that KLF5, a driver oncogene, is targeted for proteasomal degradation pathway by Fbw7.^28^ In this study, we found that PRMT5 stabilizes KLF5, further increasing its expression in lung cancer cells (Figures 3 and 4). Recent studies have shown that PRMT5 is a potential therapeutic target in different types of human cancers,^29^ including lung cancer. For instance, inhibition of PRMT5 in melanoma cells led to p53 activation by decreasing MDM4 protein expression, independent of methylation.^30^ PRMT5 inhibition also decreased the methylation of E2F1 and reduced the expression of its target genes, leading to attenuated DNA repair, cell cycle arrest and cell death.^31^ PRMT5 contributed to the disease by methylating BCL6 at R305, which is necessary for the full transcriptional repressive effects of BCL6 in lymphoma.^32^ Further, inhibition of PRMT5 in B cell lymphoma resulted in markedly upregulation of BCL6 target genes, and concomitant inhibition of both BCL6 and PRMT5 exhibited synergistic killing of BCL6‐expressing lymphoma cells. All these observations strongly indicate that controlling PRMT5 protein expression level or its enzyme activity is a promising therapeutic method to prevent human cancers, particularly lung cancer. However, we cannot ignore the vital downstream targets of PRMT5 that mediated lung cancer development.

KLF5, also known as Krüppel‐like factor 5, is a transcription factor that plays a critical role in regulating cell proliferation, differentiation and apoptosis.^33^ Abnormal expression or mutations of KLF5 have been implicated in the development and progression of several types of human cancer, including breast cancer,^5^ gastric cancer,^34^ colorectal cancer,^7^ pancreatic cancer^35^ and lung cancer.^10^ KLF5 has been shown to be upregulated and associated with poor prognosis and promotes breast cancer cell growth and invasion by activating the expression of various oncogenes and downstream signalling pathways.^36^, ^37^ Similarly, in gastric cancer, KLF5 is frequently overexpressed and has been shown to promote cell proliferation, invasion and metastasis.^38^, ^39^ In pancreatic cancer, KLF5 has been shown to be overexpressed and to facilitate cell growth and survival. KLF5 also plays a critical role in the EMT, which contributes to cancer cell invasion and metastasis.^40^ In our study, we demonstrated that KLF5 was overexpressed in lung cancer and significantly correlated with patients' survival rate (Figure 1). We also identified PRMT5 as a critical activator of KLF5 through methylation (Figures 2 and 3), which regulated the stability of KLF5 by modulating the Akt/GSK3β signalling axis (Figure 4). Our findings suggested that PRMT5 primarily controlled KLF5 expression at the post‐translational level, rather than transcriptional regulation. Importantly, PRMT5‐mediated KLF5 stabilisation induced the expression of target genes such as cyclin D1, slug and FGF‐BP1, which facilitated lung cancer cell proliferation and EMT. The evolving evidence suggested that GSK3β phosphorylated KLF5, thereby promoting Fbw7‐mediated KLF5 ubiquitination in colorectal cancer.^41^ A recent study reported that PRMT5 activated Akt by methylating arginine 15 to promote EMT and metastasis.^20^ These above observations could provide an explanation for our study, as PRMT5‐mediated KLF5 stabilisation occurs through the Akt/GSK3β signalling axis. Furthermore, our study revealed that PRMT5‐mediated KLF5 stabilisation was regulated by the proteasome rather than the lysosome signalling pathway. Interestingly, exogenous KLF5 only partially rescued lung cancer cell proliferation in PRMT5‐deficient cells, which may be due to additional roles of KLF5, such as transcriptional regulation of specific targets. Our results also demonstrated that PRMT5 was a critical epigenetic regulator for KLF5 to promote lung cancer cell growth. While exogenous KLF5 rescued the expression level in PRMT5‐deficient cells, the absence of PRMT5‐dependent KLF5 limited its ability to promote tumour growth. In addition to KLF5, PRMT5 targeted other genes, including histones such as H3R8 and H4R3,^42^, ^43^ as well as another KLF family protein, KLF4.^44^, ^45^ Further studies are needed to identify more targets of PRMT5 and its functions in lung cancer.

Collectively, our study highlights KLF5 as a new target for PRMT5 to promote lung cancer. Abnormal expression or mutations of KLF5 can contribute to the development and progression of lung cancer. Targeting PRMT5/KLF5 and its downstream signalling pathways may represent a promising therapeutic strategy for lung cancer treatment.

## AUTHOR CONTRIBUTIONS

**Hai Zhou:** Conceptualization (equal); data curation (equal); formal analysis (equal); investigation (equal); methodology (equal); resources (equal); software (equal); validation (equal); writing – original draft (equal). **Jing Chang:** Conceptualization (equal); data curation (equal); formal analysis (equal); resources (equal); software (equal). **Jingjian Zhang:** Data curation (equal); formal analysis (equal); investigation (equal); resources (equal). **Hongzhen Zheng:** Conceptualization (equal); investigation (equal); validation (equal). **Xiang Miao:** Investigation (equal); methodology (equal); writing – original draft (equal). **Huimin Mo:** Data curation (equal); software (equal). **Jie Sun:** Methodology (equal); visualization (equal). **Qin Jia:** Project administration (equal); supervision (equal); writing – original draft (equal). **Guangsheng Qi:** Conceptualization (equal); project administration (equal); supervision (equal); writing – review and editing (equal).

## CONFLICT OF INTEREST STATEMENT

The authors declared no potential conflicts of interest to this article's research, authorship and publication.

## Data Availability

The data that support the findings of this study are available on request from the corresponding author. The data are not publicly available due to privacy or ethical restrictions.
